# Edge currents shunt the insulating bulk in gapped graphene

**DOI:** 10.1038/ncomms14552

**Published:** 2017-02-17

**Authors:** M. J. Zhu, A. V. Kretinin, M. D. Thompson, D. A. Bandurin, S. Hu, G. L. Yu, J. Birkbeck, A. Mishchenko, I. J. Vera-Marun, K. Watanabe, T. Taniguchi, M. Polini, J. R. Prance, K. S. Novoselov, A. K. Geim, M. Ben Shalom

**Affiliations:** 1 School of Physics and Astronomy, The University of Manchester, Manchester M13 9PL, UK; 2 National Graphene Institute, The University of Manchester, Booth St E, Manchester M13 9PL, UK; 3 School of Materials, The University of Manchester, Manchester M13 9PL, UK; 4 Department of Physics, University of Lancaster, Lancaster LA1 4YW, UK; 5 National Institute for Materials Science, 1-1 Namiki, Tsukuba 305-0044, Japan; 6 Istituto Italiano di Tecnologia, Graphene Labs, Via Morego 30I-16163, Italy

## Abstract

An energy gap can be opened in the spectrum of graphene reaching values as large as 0.2 eV in the case of bilayers. However, such gaps rarely lead to the highly insulating state expected at low temperatures. This long-standing puzzle is usually explained by charge inhomogeneity. Here we revisit the issue by investigating proximity-induced superconductivity in gapped graphene and comparing normal-state measurements in the Hall bar and Corbino geometries. We find that the supercurrent at the charge neutrality point in gapped graphene propagates along narrow channels near the edges. This observation is corroborated by using the edgeless Corbino geometry in which case resistivity at the neutrality point increases exponentially with increasing the gap, as expected for an ordinary semiconductor. In contrast, resistivity in the Hall bar geometry saturates to values of about a few resistance quanta. We attribute the metallic-like edge conductance to a nontrivial topology of gapped Dirac spectra.

## Abstract (web summary)

The absence of a bandgap in the electronic spectrum of graphene can be overcome by breaking its lattice symmetry. The authors show that the insulating state of gapped graphene is electrically shorted by narrow edge channels exhibiting high conductivity.

The gapless spectra of mono- and bilayer graphene (MLG and BLG, respectively) are protected by symmetry of their crystal lattices. If the symmetry is broken by interaction with a substrate^1^,^2^ or by applying an electric field^3^,^4^, an energy gap opens in the spectrum. In BLG, its size *E*_gap_ can be controlled by the displacement field **D** applied between the two graphene layers. Large gaps were found using optical methods^5^ and extracted from temperature (*T*) dependences of resistivity *ρ* at sufficiently high *T* (refs 6, 7, 8, 9, 10). Their values are in good agreement with theory. On the other hand, at low *T* (typically, below 50 K), *ρ* at the charge neutrality point (CNP) in gapped graphene is often found to saturate to relatively low values that are incompatible with large *E*_gap_ (refs 6, 7, 8, 9, 10, 11). This disagreement is attributed to remnant charge inhomogeneity^6^,^8^,^10^ that results in hopping conductivity and, therefore, weakens *T* dependences. Alternative models to explain the subgap conductivity were proposed, too. They rely on the nontrivial topology of Dirac bands in gapped MLG and BLG^12^,^13^,^14^,^15^, which gives rise to valley-polarized currents^13^,^14^,^15^. Large nonlocal resistances were reported for both graphene systems at the CNP and explained by valley currents propagating through the charge-neutral bulk^16^,^17^,^18^. Graphene edges^12^,^15^, p–n junctions^19^ and stacking boundaries^20^ can also support topological currents. These conductive channels were suggested to shunt the insulating bulk, leading to a finite *ρ*. Experimentally, the situation is even more complicated because additional conductivity may appear for trivial reasons such as charge inhomogeneity induced by chemical or electrostatic doping^21^,^22^,^23^. Here we show that highly conductive channels appear near edges of charge-neutral graphene if an energy gap is opened in its spectrum. We tentatively attribute the edge channels to the presence of such unavoidable defects as, for example, short zigzag-edge segments^12^. Their wavefunctions extend deep into the insulating bulk where they sufficiently overlap to create a quasi-one-dimensional impurity band with little intervalley scattering and high conductivity. We believe that, in certain graphene devices, the localization length can be very long, comparable to typical distances between electric contacts, which effectively results in shunting the gapped bulk.

## Results

### Josephson current distributions

**Figure 1:**
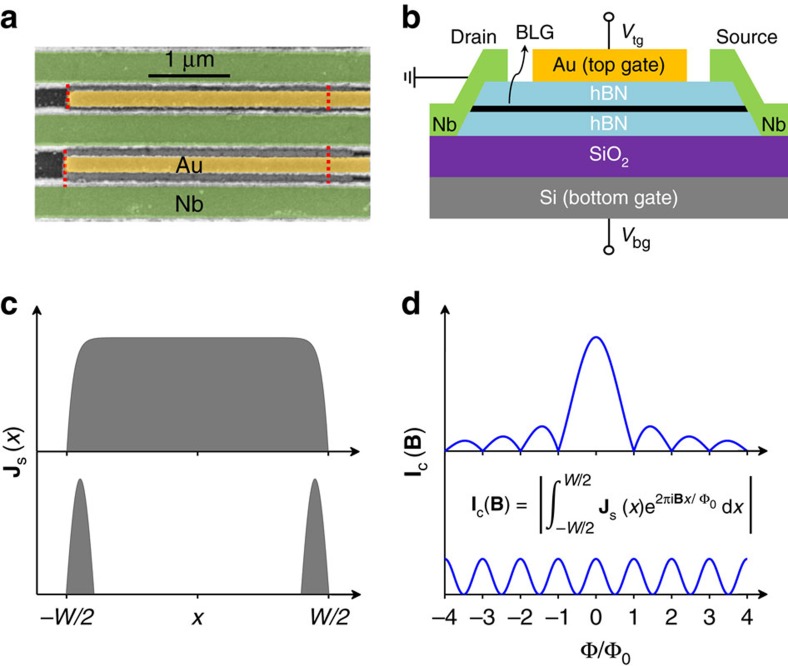
Gated Josephson junctions and spatial distribution of supercurrents. (**a**) Electron micrograph of our typical device (in false colour). Nb leads (green) are connected to bilayer graphene (its edges are indicated by red dashes). The top gate is shown in yellow. (**b**) Schematics of such junctions. (**c**) Illustration of uniform and edge-dominant current flow through Josephson junctions (top and bottom panels, respectively). (**d**) The corresponding behaviour of the critical current **I**_c_ as a function of **B**. **I**_c_(**B**) is related to **J**_s_(*x*) by the equation shown in **d**. For a uniform current flow, **I**_c_ should exhibit a Fraunhofer-like pattern (top panel) such that the supercurrent goes to zero each time an integer number *N* of magnetic flux quanta Φ_0_ thread through the junction. Maxima in **I**_c_ between zeros also become smaller with increasing *N*. For the flow along edges (bottom panel), **I**_c_ is minimal for half-integer flux values Φ=(*N*+1/2)Φ_0_, and maxima in **I**_c_ are independent of **B**. The spatial distribution **J**_s_(*x*) can be found^24^,^25^ from **I**_c_(**B**) using the inverse FFT. Due to a finite interval of Φ over which the interference pattern is usually observed experimentally, **J**_s_(*x*) obtained from the FFT analysis are usually smeared over the *x* axis as shown schematically in **c**.

We start with discussing behaviour observed for superconductor-graphene-superconductor (SGS) Josephson junctions. Our devices were short and wide graphene crystals that connected superconducting Nb electrodes^24^ (Fig. 1). Each device contained several such SGS junctions with the length *L* varying from 300 to 500 nm and the width *W* from 3 to 5 μm. To ensure highest possible quality^24^, graphene was encapsulated between hexagonal boron nitride (hBN) crystals with the upper hBN serving as a top gate dielectric and the Si/SiO_2_ substrate as a bottom gate (Fig. 1b). For details of device fabrication and characterization, we refer to ‘Methods' section and Supplementary Note 1 and Supplementary Fig. 1. By measuring the critical current **I**_c_ as a function of perpendicular magnetic field **B**, the local density **J**_s_(*x*) in the *x* direction perpendicular to the supercurrent flow can be deduced^25^, as illustrated in Fig. 1c,d. This technique is well established and was previously used to examine, for example, edge states in topological insulators^26^ and wave-guided states in graphene^22^. In our report, we exploit the electrostatic control of the BLG spectrum to examine how **J**_s_(*x*) changes with opening the gap.

**Figure 2:**
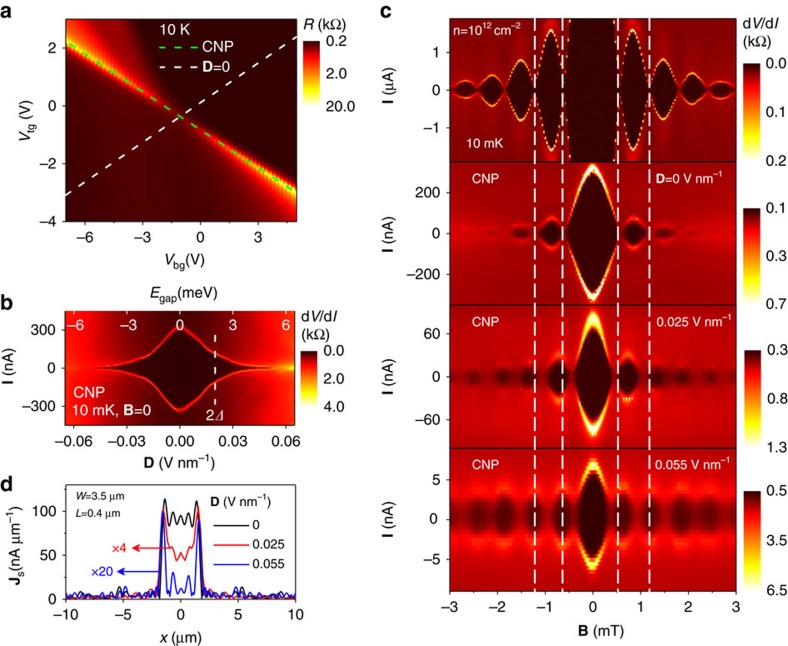
Redistribution of supercurrent as the gap opens in bilayer graphene. (**a**) Resistance *R* of one of our Josephson junctions (3.5 μm wide and 0.4 μm long) above the critical *T* as a function of top and bottom gate voltages. The dashed white line indicates equal doping of the two graphene layers with carriers of the same sign. The dashed green line marks the CNP (maximum *R*) and indicates equal doping with opposite sign carriers. (**b**) Differential resistance *dV*/*dI* measured along the green line in **a** at low *T* and in zero **B**. Transition from the dissipationless regime to a finite voltage drop shows up as a bright curve indicating **I**_c_. The vertical line marks the superconducting gap of our Nb films. (**c**) Interference patterns in small **B**. The top panel is for the case of high doping [**I**_c_(**B**=0) ≈10 μA] and indistinguishable from the standard Fraunhofer-like behaviour illustrated in Fig. 1d. The patterns below correspond to progressively larger *E*_gap_. Changes in the phase of Fraunhofer oscillations are highlighted by the vertical dashed white lines. (**d**) Extracted spatial profiles of the supercurrent density (**J**_s_) at the CNP for the three values of **D** in **c**.

By varying the top and bottom gate voltages (*V*_tg_ and *V*_bg_, respectively), it is possible to keep BLG charge neutral while doping the two graphene layers with carriers of the opposite sign (see Fig. 2a). This results in the displacement field **D**(*V*_tg_,*V*_bg_) that translates directly into the spectral gap^3^,^4^,^5^,^6^. Its size *E*_gap_(**D**) can be deduced not only theoretically but also measured experimentally, as discussed in section 1 of Supplementary Information Note 1. To quantify proximity superconductivity in our devices, we define their critical current **I**_c_ as the current at which the differential resistance d*V*/d*I* deviates from zero above our noise level^24^. With reference to Fig. 2b, **I**_c_ corresponds to the edge of the dark area outlined by bright contours. At high doping (Fermi energy >*E*_gap_) and low *T*, **I**_c_ is found to depend weakly on **D**, reaching values of a few μA μm^−1^, in agreement with the previous reports^22^,^24^,^27^. The supercurrent generally decreases with increasing junction's resistance and becomes small at the CNP. Its value depends on *E*_gap_ (Fig. 2b). Accordingly, the largest **I**_c_ in the neutral state is found for zero **D** (no gap) reaching ≈300 nA for the junction shown in Fig. 2. The value drops to 2 nA at **D***=*±0.07 V nm^−1^, which corresponds to *E*_gap_≈7 meV. For larger gaps, **I**_c_ becomes smaller than 1 nA and could no longer be resolved because of a finite temperature (down to 10 mK) and background noise^24^.

We analyse changes in the interference pattern, **I**_c_(**B**), with increasing **D** (that is, increasing *E*_gap_). At zero **D**, we observe the standard Fraunhofer pattern at the CNP, which is basically similar to that measured at high doping (cf. two top panels of Fig. 2c). Only absolute values of **I**_c_ are different because of different *ρ*, as expected^24^. The Fraunhofer pattern corresponds to a uniform current flow (Fig. 1c,d). In contrast, the interference pattern measured at the CNP for a finite gap is qualitatively different (see Fig. 2c; **D**=0.055 V nm^−1^). The phase of the oscillations changes by 90° and the central lobe becomes twice narrower. In addition, the side lobes no longer decay with increasing **B** but exhibit nearly the same amplitude (additional example in Supplementary Note 2 and Fig. 2). Such a pattern resembles the one shown schematically in Fig. 1d for the case of the supercurrent flowing along edges. The only difference with Fig. 1d is that in our case the central lobe remains higher than the others. For quantitative analysis, we calculated the inverse fast Fourier transform (FFT) of **I**_c_(**B**), which yielded^26^ the current distributions **J**_s_(*x*) shown in Fig. 2d. The supercurrent is progressively pushed towards device edges with increasing the gap. This is already visible for **D***=*0.025 V nm^−1^ but further increase in **D** suppresses the bulk current to practically zero, within the experimental accuracy of our FFT analysis (Fig. 2d and Supplementary Fig. 3). The accuracy is limited by a finite range of **B** in which the interference pattern could be detected (Supplementary Note 3).

**Figure 3:**
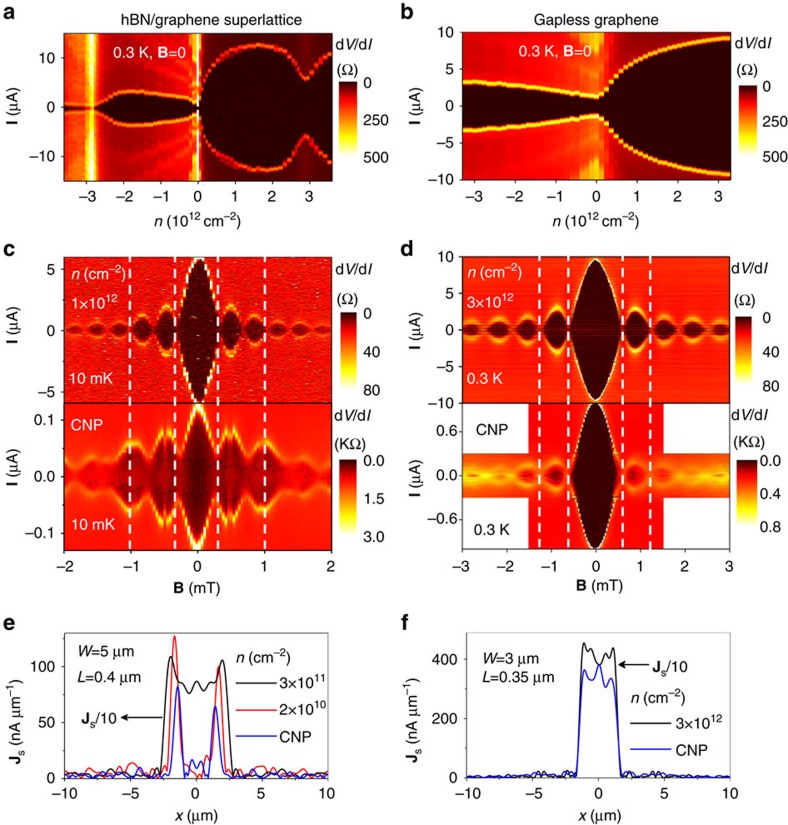
Interference patterns and supercurrent flow in gapped and non-gapped graphene monolayers. (**a**) Differential resistance as a function of carrier concentration *n* and applied current *I* for a Nb-MLG-Nb junction (5 μm wide and 0.4 μm long). The gap is induced by alignment with the bottom hBN crystal. (**b**) Same for encapsulated but nonaligned monolayer graphene (the junction is 3 μm wide and 0.35 μm long). (**c**) Interference patterns for gapped MLG at relatively high doping (top panel) and at the CNP. (**d**) Same for non-gapped graphene. (**e**,**f**) Corresponding spatial profiles of the supercurrent density (**J**_s_). They were calculated using experimental patterns such as shown in **c**,**d**. Note that graphene edges in **e** support fairly high supercurrent at the CNP, whereas there is no indication of any enhanced current density along edges for the non-gapped case in **f**.

For completeness, we have also studied SGS junctions that were fabricated using monolayer graphene placed on top of hBN and aligned along its crystallographic axes. Such alignment (within 1–2°) results in opening of a gap of ≈30 meV at the main CNP^1^,^2^, and secondary CNPs appear for high electron and hole doping^1^,^2^,^16^. Unlike for the case of BLG, *E*_gap_ cannot be changed *in situ* in MLG devices, but one can still compare interference patterns for neutral and doped states of the same SGS junction and, also, use nonaligned junctions as a reference. Figure 3a,b shows typical behaviour of **I**_c_ as a function of carrier concentration *n* for SGS devices made from gapped (aligned) and gapless (nonaligned) MLG. In the gapped device, the supercurrent is suppressed not only at the main CNP but also at secondary CNPs. For all electron and hole concentrations away from the CNPs, both devices exhibit the standard Fraunhofer pattern indicating a uniform supercurrent flow (cf. top panels of Fig. 3c,d). The same is valid at the CNP in gapless graphene (Fig. 3d,f). In contrast, for gapped MLG, the interference pattern at the main CNP undergoes significant changes such that the phase and period of oscillations in **I**_c_ change (Fig. 3c; bottom panel), somewhat similar to the behaviour of gapped BLG at the CNP. Quantitative analysis using FFT again shows that, in gapped MLG, the supercurrent flows predominantly along graphene edges for *n*<±5 × 10^10^ cm^−2^ (Fig. 3e). The figure seems to suggest a shift of conductive channels from edges into the interior. This shift originates from the increase in the Fraunhofer period at the CNP in Fig. 3c and corresponds to a decrease in the junction's effective area. However, we believe that this shift may arise from non-uniform doping along the current direction. Our MLG devices do not have a top gate and this allows doping by metal contacts to extend significantly (tens of nm) inside the graphene channel^28^, which reduces the effective length of the junction.

We emphasize that the observed redistribution of supercurrents towards edges is an extremely robust effect observed for all eight gapped graphene junctions we studied and in none without a gap (more than 10)^24^. In principle, one can imagine additional electrostatic and/or chemical doping near graphene edges^21^,^22^,^23^ (Supplementary Note 4), which would enhance their conductivity and, hence, favour local paths for supercurrent. This mechanism disagrees with the fact that edge supercurrents appeared independently of the CNP position as a function of gate voltage (residual doping in our devices varied from practically zero to <10^11^ cm^−2^) and were observed for devices with the top gate being only a few nm away from the graphene plane. The latter facilitates a uniform electric field distribution (Supplementary Fig. 4). Chemical doping at graphene edges was previously reported in non-encapsulated^21^ and, also, encapsulated but not annealed devices^23^. All our devices were encapsulated and thoroughly annealed, and some of them had edges that were fully covered by top hBN rather than exposed to air (Supplementary Figs 5 and 6). We also note our Josephson experiments yielded similar supercurrent densities at BLG edges, even in the case where the two edges were fabricated differently (one is etched as discussed above and the other cleaved and covered with hBN; see Fig. 1a). The latter observation in particular indicates little external doping along the edges. Importantly, we have found no evidence for enhanced transport along edges of similar but gapless graphene devices. To this end, we refer, for example, to Fig. 3e,f. In the gapped MLG device, near-edge **J**_s_ reaches ≈100 nA μm^−1^. Such supercurrents would certainly be visible in the distribution profile of the non-gapped graphene at the CNP in Fig. 3f. All the above observations point at a critical role of the presence of the gap in creating local edge currents.

### Corbino geometry

**Figure 4:**
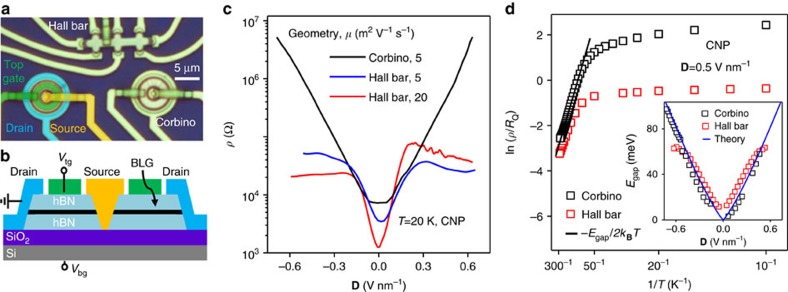
Charge-neutral bilayer graphene in the Corbino and Hall bar geometries. (**a**) Optical image of one of our devices with a Hall bar and two Corbino disks. The left-disk image is coloured to indicate source, drain and top gate electrodes. (**b**) Cross-sectional schematic of our double-gated Corbino devices. (**c**) Resistivity *ρ* at the CNP for Corbino and Hall bar geometries as a function of **D**. For the Corbino device, *ρ* changes exponentially over three orders of magnitude. The Hall bars exhibit saturation to a few *R*_*Q*_. (**d**) Arrhenius plot for *ρ*(*T*). The energy gap *E*_gap_ is calculated from the linear slopes at *T*>100 K, which are similar for both Corbino and Hall bar geometries. Below 50 K, the Hall bar device exhibits little *T* dependence. Inset: *E*_gap_ found for various **D** (symbols). The blue curve is tight-binding calculations for the BLG gap from ref. 3.

While providing important insights about the current flow, Josephson interference experiments are limited to small *E*_gap_ such that junction's resistance remains well below 1 MOhm allowing superconducting proximity. To address the situation for the larger gaps accessible in BLG devices, we compare their normal transport characteristics in the Corbino and Hall bar geometries. Because the Corbino geometry does not involve edges, such a comparison has previously been exploited to investigate the role of edge transport (for example, in the quantum Hall effect^29^). A number of dual-gated BLG devices such as shown in Fig. 4a were fabricated and examined over a wide range of **D** and *T*. Our experiments revealed a striking difference between *ρ* measured in the two geometries. In the Corbino geometry, *ρ* at the CNP rises exponentially with **D** and its value is limited only by a finite dielectric strength of ≈0.7 V nm^−1^ achievable for our hBN (Fig. 4b) and, at low *T*, by leakage currents. In contrast, in the Hall bar geometry, *ρ* at the CNP saturates at **D** as low as <0.2 V nm^−1^, reaching only a few tens of kOhms at all *T* (Fig. 4c). This disparity in the behaviour of the Hall bar and Corbino devices was observed under the same measurement conditions and despite the same or higher homogeneity in the former devices (Supplementary Note 5 and Supplementary Fig. 7). The profound difference unambiguously points at a finite conductivity caused by the presence of graphene edges, in agreement with the conclusions achieved from our Josephson experiments.

Another noteworthy distinction between the two geometries is their temperature dependences at the CNP. For *T* above 100 K, both Corbino and Hall bar devices exhibited the same activation behaviour *ρ*∝exp(*E*_gap_/2*k*_*B*_*T*) as expected for a semiconductor with the gap *E*_gap_ (Fig. 4d). Our measurements over a wide range of **D** yielded *E*_gap_[meV]≈100 × **D**[V nm^−1^], in quantitative agreement with theory and previous reports^5^ (inset of Fig. 4d). At lower *T*, resistivity of the Corbino devices continued growing and is well described by hopping conductivity that may involve both nearest neighbour and variable range hopping^6^,^8^,^9^,^10^ (Fig. 4d). On the other hand, *ρ*(*T*) found using the Hall bars rapidly saturated below 100 K to values of a few resistance quantum *R*_*Q*_=*h*/e^2^ and changed little (by <30%) with decreasing *T* down 2 K. The saturation behaviour is similar to that observed for conductance along a p–n junction in oppositely biased BLG^19^, and along walls separating BLG domains with AB and BA stacking^20^.

## Discussion

Two possible scenarios for shunting the insulating state of gapped graphene have previously been put forward. Both rely on nontrivial topology of the gapped Dirac spectrum. One of them considers electronic states due to short zigzag segments^15^ that may be present even at relatively random edges^12^. Although these states decay exponentially into the gapped bulk, their penetration length *ξ* is very long with respect to the lattice constant *a*. For MLG and BLG, *ξ* can be estimated as ≈*ħv*/*E*_gap_ and *ħ*/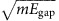, respectively, where *ħ* is the reduced Planck constant, *v* the Fermi velocity in MLG and *m* the effective mass in BLG. For our typical gaps, *ξ* is about 10–20 nm, much larger than *a*. This suggests that wavefunctions of isolated zigzag states should strongly overlap inside the bulk creating a quasi-one-dimensional band. Moreover, because *ξ*/*a*≫1, the wavefunctions mostly reside in the bulk where there are little defects, which ensures that impurity bands are effectively protected against backscattering. The situation resembles the modulation doping used to achieve high-carrier mobilities in semiconductor quantum wells. The observed saturation of *ρ* to ∼*R*_*Q*_ and the long-range nonlocal resistance reported previously^16^,^17^,^18^ imply that the mean free path along the quasi--one-dimensional channels can reach a micrometre scale for high-mobility graphene. Although numerical simulations^12^ yielded zero-*T* localization lengths at least an order of magnitude shorter than this scale, localization in the edge channels may be suppressed by a finite *T* and electron–electron interactions that are prominent especially in low-dimensional conductors. Such delocalization effects have so far not been investigated theoretically. The invoked edge channels would be consistent with our experimental observations. Obviously, the mean free path can vary from sample to sample and strongly depend on fabrication procedures, which may explain only weakly saturating behaviour that was reported in some gapped graphene devices^16^,^17^,^19^. In addition, there is a complementary scenario that also relies on the nontrivial topology of the gapped Dirac spectra but may not require zigzag segments. The valley Hall effect is inherent to gapped graphene and generates valley currents that flow perpendicular to applied electric field^13^,^16^. If injected from electric contacts into the gapped bulk, they are expected to become squeezed towards weakly conductive edges, similar to what is known for the case of the quantum Hall effect^30^ and in agreement with recent simulations for gapped MLG^14^. Lastly, let us mention another relevant suggestion that a weak confining potential at graphene edges may guide electronic states over large distances, independently of its strength^22^,^31^. In the latter scenario, an enhanced edge conductance is expected irrespectively of the gap size, which seems to contradict our experimental observation that there is little enhancement of near-edge supercurrent in the absence of the gap.

Our results can explain low apparent resistivity often observed for charge-neural gapped graphene at low temperatures, especially in devices made from high-quality graphene in which the bulk is expected to contribute little to either hopping conductivity or backscattering of edge modes^5^,^6^,^7^,^8^,^9^,^10^,^11^,^19^. Further experiments and theory are needed to distinguish between the possible scenarios described above and elucidate the nature of the reported edge conductance.

## Methods

### Device fabrication

Mono- or bilayer graphene crystals were encapsulated between hBN crystals (typically, ≈30 nm thick) using the dry transfer technique as detailed previously^32^. The hBN-graphene-hBN stack was assembled on top of an oxidized Si wafer (300 or 90 nm of SiO_2_) and then annealed at 300 °C in a forming gas (Ar–H_2_ mixture) for 3 h. As the next step, we used the standard electron-beam lithography to create a poly (methyl methacrylate) (PMMA) mask that defined contact regions. Reactive ion etching (Oxford Plasma Lab 100) was employed to make trenches in the heterostructure through the mask. We used a mixture of CHF_3_ and O_2_, which provided easy lift-off of PMMA, so that metal contacts could be deposited directly after plasma etching. This also allowed us to minimize contamination of the exposed graphene edges^24^. After this, for BLG devices, another metal film (typically, Au/Cr) was deposited on top of the heterostructure to serve as the top gate. To avoid the edges of graphene extending out of the metal gate, the latter is used as a part of the final etch-mask; the uncovered graphene between the contacts and the gate is protected by a second PMMA mask, allowing the metal gate to extend slightly at the crucial edge locations. For the Hall bar geometry, we often used an additional hBN crystal to cover the hBN-graphene-hBN stack after plasma etching, which allowed the metal film for the top gate to go over exposed graphene edges without touching them. To provide the central contact in Corbino devices, we used air bridges^33^. In some of our Josephson devices, graphene was not etched but made directly from cleaved crystals selected to have a strip-like shape. In this case, graphene edges were not exposed but fully encapsulated in hBN. Similar transport and Josephson behaviour was found in all cases, independent of the variations in fabrication procedures.

### Transport experiments

All electrical measurements were carried out in a He3 cryostat (Oxford Instruments) for *T* down to 0.3 K and, for lower *T*, in a dilution refrigerator with the base temperature of 10 mK (BlueFors Cryogenics). The differential resistance was measured in a quasi-four-terminal configuration (two superconducting leads for driving the current and the other two for measuring voltage) using a low-frequency lock-in technique. All electrical connections to our devices passed through a cold RC filter (Aivon Therma) placed close to the sample and additional AC filters were used outside the cryostats. At large displacement fields, our Corbino devices exhibited high resistivity such that the lock-in technique became inappropriate. In this case, we used dc measurements.

### Data availability

The data that support the findings of this study are available from the corresponding author upon request.

## Additional information

**How to cite this article:** Zhu, M. J. *et al*. Edge currents shunt the insulating bulk in gapped graphene. *Nat. Commun.* **8,** 14552 doi: 10.1038/ncomms14552 (2017).

**Publisher's note**: Springer Nature remains neutral with regard to jurisdictional claims in published maps and institutional affiliations.

## Supplementary Material

Supplementary Information Supplementary Figures, Supplementary Notes and Supplementary References
