# Supplementary material for: Edge currents shunt the insulating bulk in gapped graphene
Source: Nat Commun. 2017 Feb 17;8:14552. doi: 10.1038/ncomms14552 (PMC5321719; doi:10.1038/ncomms14552)
Supplement: Supplementary Information — Supplementary Figures, Supplementary Notes and Supplementary References [file ncomms14552-s1.pdf]

Supplementary Information

Supplementary Note 1, Characterization of double gated bi-layer graphene.

By implementing top and bottom gate electrodes in the studied devices, it is possible control the charge carrier density n and the electric displacement field **D** between the two layers independently[1]. Below, the calibration procedure of $n(V_{tg},V_{bg})$ and $\mathbf{D}(V_{tg},V_{bg})$ (V_{tg} and V_{bg} are the top and bottom gate voltages respectively). Examples of such measurements for Hall-bar, Josephson junction, and Corbino device geometries are presented in Supplementary Figure 1.

At first, the resistance R is plotted as a function of the two gates (Supplementary Figure 1a,c,d). The sharp peak in R determines the position of the charge neutrality point (CNP), Supplementary Figure 1b. The axis parallel to the charge neutrality line is determined (see black arrows, Supplementary Figure 1a), and its slope: $\Delta V_{bg}/\Delta V_{tg}\approx 2$ is equal to the capacitance ratio of the two gates C_{tg}/C_{bg}. Smaller C_{bg} is expected for the thicker SiO_2 dielectric at the bottom, and requires separate characterization for each device due to the different thickness of hBN which we place on top of SiO_2. The negative slope ($\Delta V_{bg}/\Delta V_{tg}\approx$-2, marked by white line on Supplementary Figure 1a), corresponds to adding the same charge to both layers and changing the total n while keeping a fixed **D**. In order to accurately measure n we analyze the quantum oscillations in R at high magnetic fields and away from CNP. From this, the capacitance (per unit of area) for each gate is extracted using: $ne = C_{tg}\Delta V_{tg} + C_{bg}\Delta V_{bg}$, and the displacement field is calculated to be: $\mathbf{D} = (C_{tg}\Delta V_{tg} - C_{bg}\Delta V_{bg})/2\varepsilon_0$.

The energy gap E_{gap} is measured independently from the Arrhenius-like activation of R at high temperatures as shown in the main text, Fig. 4d. When measured at different **D**, we find E_{gap}[meV]$\approx 100\times$**D**[V·nm^{-1}] to hold for all our BLG devices (see inset to Fig. 4d), in agreement with previous reports[2] and calculations[3].

The devices presented here also show saturation of the sub-gap R with increasing **D** in the Hall bar and Josephson geometry, and exponentially increasing R in the Corbino geometry (Supplementary Figure 1b,d). For the latter, an additional increase in R is observed at a fixed value of the bottom gate $V_{bg}\approx$-3 V (Fig. S1c). It corresponds to the CNP of the BLG at the locations in the device not covered by the top gate (see image in Fig. 4a, main text). This spatial separation of the top gate from the metal-graphene interface guarantees a negligibly-low contact resistance at high **D** for the two-probe measurement in this geometry.

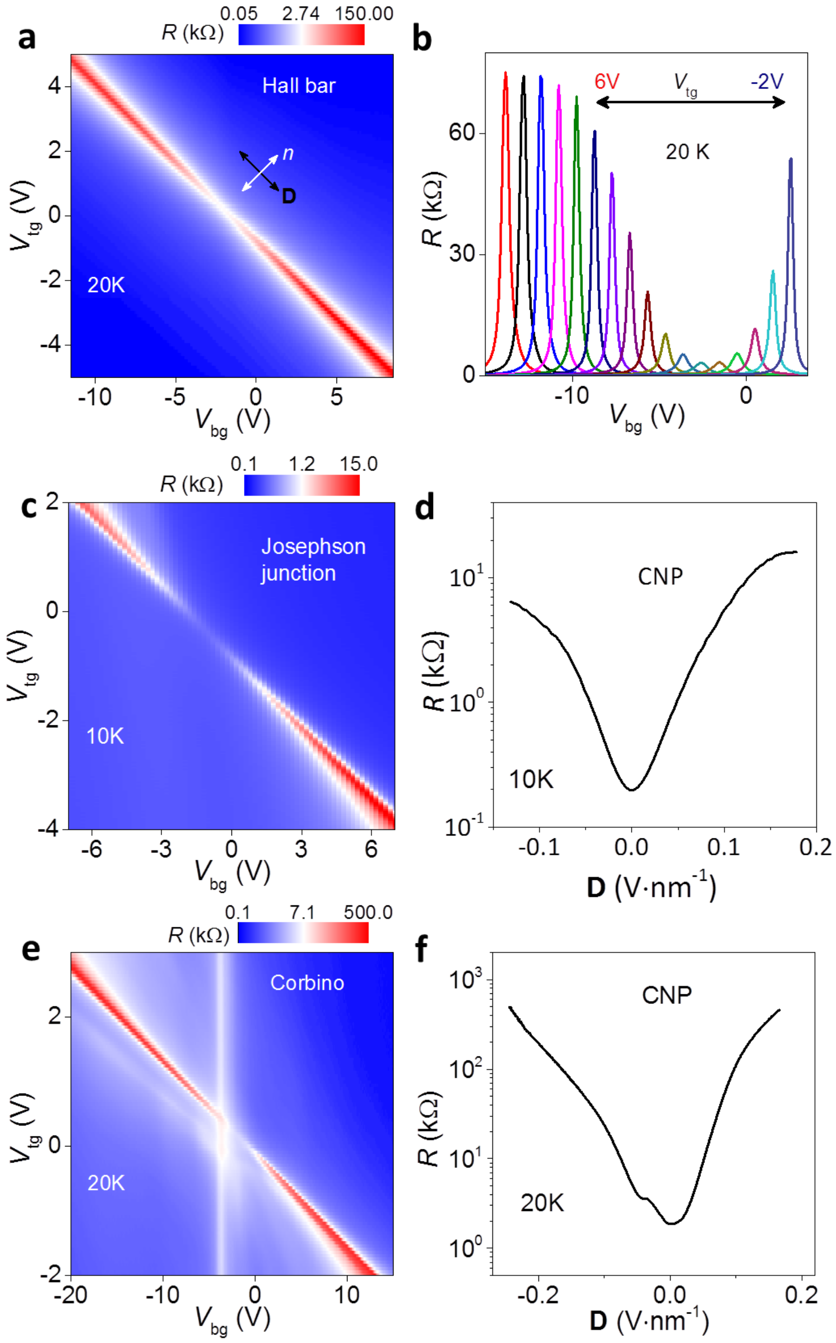

Supplementary Figure 1│ Characterization of double-gated bilayer graphene. a, Color-plots of the resistance R (in log-scale) as a function of the top and bottom gate voltages, for the Hall-bar geometry (the measured section is 2.3 µm wide and 6.6 µm long). **b,** Bottom gate scans from the map in (a) at different fixed values of the top gate. At the charge neutrality point (CNP) *R* is saturated for V_{tg}>5V corresponding to **D** ≈-0.2V·nm^{-1} as shown in the main text (Fig. 4c). **c,** Color-plots of the resistance *R* (in log-scale) as a function of the top and bottom gate voltages for the Josephson junction studied in the main text Fig. 2. **d,** Resistance at CNP extracted from the map in (c). The increase in *R* is saturated for displacement field **D** ≈0.15V·nm^{-1}. **e,** Color-plots of the resistance *R* (in log-scale) as a function of the top and bottom gate voltages for a Corbino "edge-less" device. Here the top gate is 10 µm wide and 1 µm long, and it is separated by 1 µm from the inner and outer contacts. The vertical white line at V_{bg}≈-3V corresponds to the charge neutrality point in the part of the device which is not covered by the top gate. **f,** Resistance at CNP extracted from the map in (c). The increase in *R* is exponential with the displacement field **D**.

Supplementary Note 2, Example of additional BLG Josephson junction.

Here we present a second dataset for an additional BLG Josephson junction. As the gap opens, redistribution of supercurrent is observed, Supplementary figure 2, in a similar way to the junction studied in the main txt, Fig. 2.

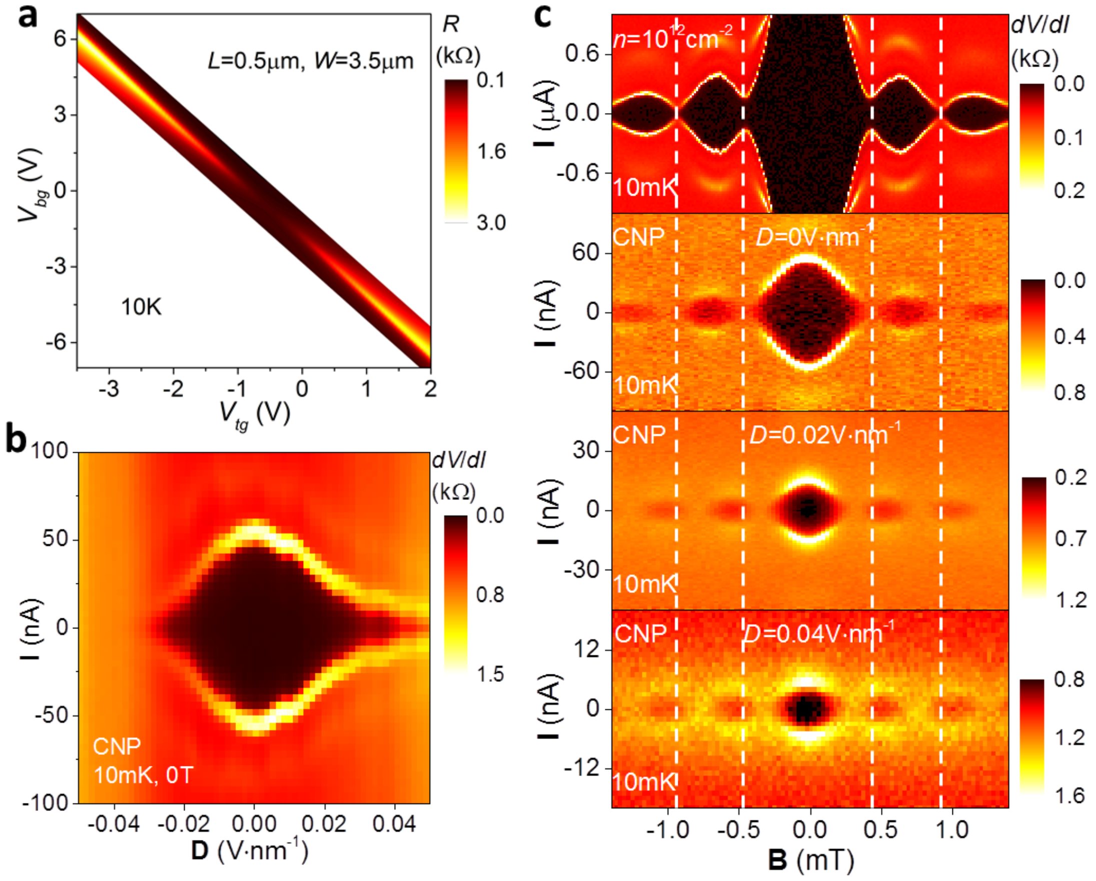

Supplementary Figure 2│ Redistribution of supercurrent as the gap opens in bilayer graphene. a, Resistance *R* of a Josephson junctions (3.5 μm wide and 0.5 μm long) above the critical T as a function of top and bottom gate voltages. **b,** Differential resistance *dV/dI* measured along the CNP line in **a** at low *T* and in zero **B**. Transition from the dissipationless regime to a finite voltage drop shows up as a bright curve indicating $\mathbf{I}_c$. **c,** Interference patterns in small **B**. The top panel is for the case of high doping [$\mathbf{I}_c$(**B** =0) ≈2 μA] and indistinguishable from the standard Fraunhofer-like behavior illustrated in Fig. 1d. The patterns below correspond to progressively larger E_{gap}. Changes in the phase of Fraunhofer oscillations, consistent with the formation of edge modes, are highlighted by the vertical dashed white lines.

Supplementary Note 3, Bulk versus edge distribution of the supercurrent in bi-layer graphene.

In this section we further analyze the interference patterns of the supercurrent $\mathbf{I}_c(\mathbf{B})$ at CNP for different values of **D**. The inverse FFT is calculated to extract the local current distribution $\mathbf{J}_s(x)$ (see Supplementary Figure 3b and Fig. 2d of the main text). Then the current density at the edges is compared to the one at the center of the junction. We find that the transition from uniform current distribution to the edge dominant flow is rather sharp and occupies the range in the displacement $0.015<\mathbf{D}<0.03$ V·nm^{-1} (see Supplementary Figure 3b). The bulk component of $\mathbf{J}_s$ is dramatically reduced above $\mathbf{D}\approx 0.03$ V·nm^{-1} and the supercurrent becomes restricted to the edge channels. To this end we note that the supercurrent in the graphene is carried by Andreev pairs coupled by the superconductor gap Δ. At zero temperature and for entirely gaped graphene, finite $\mathbf{I}_c$ is not expected for $E_{gap} > 2\Delta$ because tunneling processes are improbable across this 400nm long barrier (the length of the graphene channel). The analysis of $\mathbf{J}_s(x,\mathbf{D})$ below suggest that the cut-off for the bulk contribution is indeed happening at $E_{gap} \approx 2\Delta$ (=2meV in the case of these Nb contacts[4]). Thus the finite $\mathbf{I}_c$ at the edges persisting to large gaps indicates that the edges are less gapped than the bulk, or not gapped at all.

The inverse FFT shown in Supplementary Figure 3b and Fig. 2d can be fitted by Gaussians in order to estimate the width w to which the edge mode extend into the bulk (taken as the width of the peak at half maximum). Yet a limit on the spatial resolution of $\mathbf{J}_s(x)$ arise, which can be defined by the largest number of the magnetic flux in which the interference pattern $\mathbf{I}_c(\mathbf{B})$ still can be detected (additional limitation of the calculation is the assumption of a sinusoidal current-phase relation, which is not accurate in these long and ballistic Josephson junctions). We can reliably extract the interference over ≈10 periods (flux quanta) before the noise level or other ballistic effects[4] alters its pattern. This number correspond to a spatial resolution limit of $\approx W/10=350$ nm for the studied junctions of the width W. The calculated w from the FFT is 650nm and 450nm at $\mathbf{D}=0.025$ V·nm^{-1} and 0.055 V·nm^{-1} respectively and should be regarded as an upper limit of the width of the edge channels.

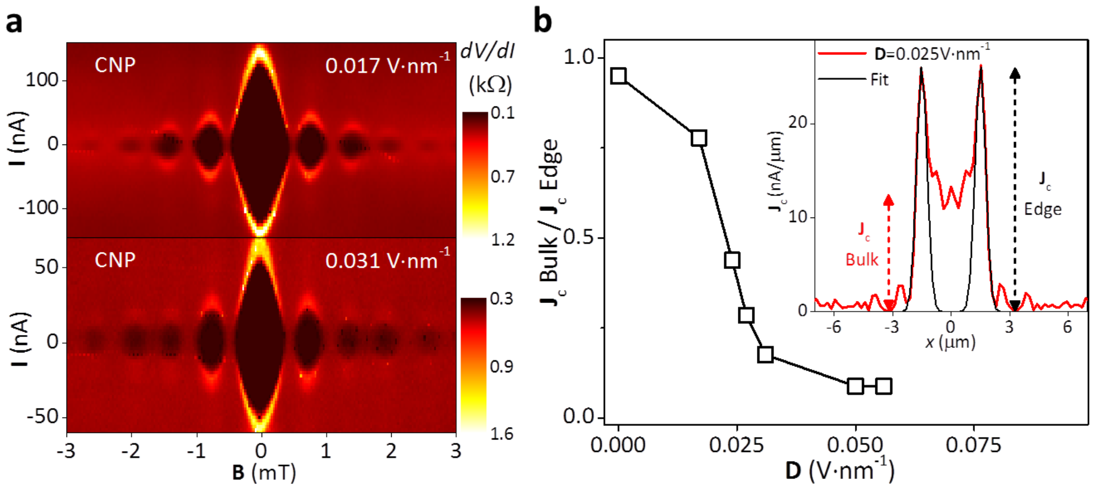

Supplementary Figure 3│ Supercurrent distribution as a function of the displacement field D. a, Examples of interference patterns measured at two different **D**. **b,** The supercurrent density at the bulk and at the edges is extracted from inverse FFTs of the $\mathbf{I}_c(\mathbf{B})$ patterns (as shown in Fig. 2d). The transition from bulk dominant to edge current is sharp, in the range $\mathbf{D}\approx 0.015$ to 0.03 V·nm^{-1}. Inset, Gaussian fit to the edge current distribution.

Supplementary Note 4, Chemical and electrostatic doping at the edge.

In principle, external doping near graphene edges may offer an alternative explanation for edge-transport when the bulk is gaped. In the following we consider various doping scenarios, how to minimize their effect and how to test its presence experimentally. The three doping scenarios are: i) Chemical variations at the edge, which may depend significantly on the fabrication process[5]. To minimize its effect we anneal all samples as an essential part of our fabrication procedures. ii) Electrostatic doping arising from a finite separation between the gate electrodes and graphene[6]. The spatial range of this stray doping is determined by the distance to the gates, which for this reason were fabricated as close as possible to the graphene plane (≈30 and 120nm away for the top and bottom gates, respectively). iii) Non-uniform termination of the two layers in the BLG. This is avoided by dry etching the two layers simultaneously using a highly anisotropic etching process.

To evaluate the effect of external doping, we measured devices in which the two edges of the Josephson junction were different (see Fig. 1a, Supplementary Figure 4). One edge of the BLG is encapsulated by hBN and overlaid by the top gate, while at the other edge the top gate terminates and the BLG edge is uncovered. In principle, the different profiles should result in different chemical and electrostatic doping. Calculations of the electrostatic doping profiles are shown for the two edge configurations (Supplementary Figure 4). When the top gate terminates above the graphene edge, the charge density accumulation is diverging near it, with a lateral cutoff given by the thickness of the dielectric spacer. 100 nm away from the edge, the carrier density is expected to be ≈5×10^{9} cm^{-2} for 1V / 0.24V applied to the bottom / top gate, respectively (corresponding to $\mathbf{D}$≈0.03$V\cdot nm^{-1}$). In contrast, the configuration of extended gate and hBN show negligible electrostatic doping. The effect of electrostatic or chemical doping, if significant, should clearly favor edge conductance along one of the edges only.

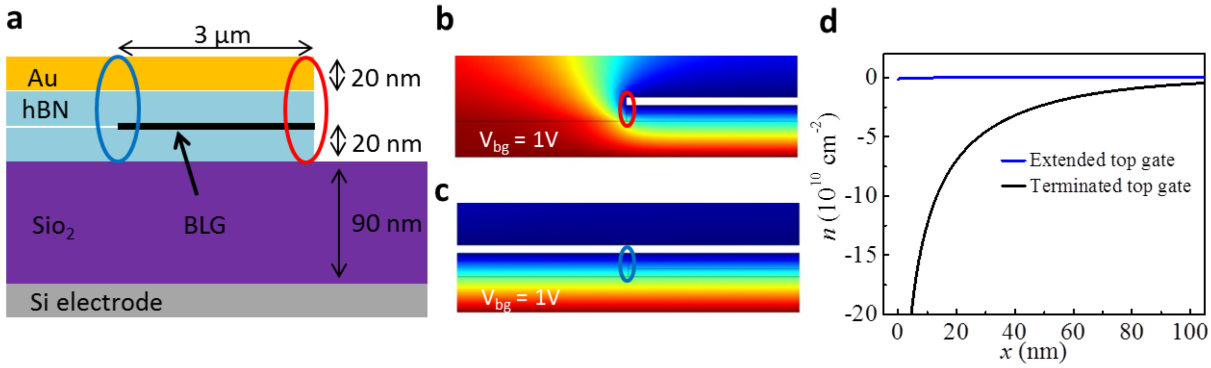

Supplementary Figure 4│ Electrostatic modeling of edge doping. a, Schematic cross-section of a Josephson junction with different edge profiles (the other cross-section and top view are shown in figure 1 a,b main text). **b, c,** Finite element calculation of the electrostatic potential distribution for the two edge configurations. The bottom gate is fixed at 1 Volt while the top gate is tuned to fix a zero potential at the bulk of the BLG (colored circles mark the two edge configurations) **d,** Calculated carrier density accumulation as function of the distance from the edge for the extended (Blue curve) and edge-terminated (red) profiles. For the former, charge accumulation is negligible.

Here we point out the high sensitivity of the supercurrent interference patterns to asymmetric supercurrent density distribution. Conceptually this sensitivity can be described as follow: if the maximum supercurrent density in the two edges is precisely equal, flux penetration can force it in opposite directions for each edge, such that a zero net supercurrent can be driven across the

junction (the measured $\mathbf{I}_c$). On the other hand, uneven critical current density will preserve a finite "net" supercurrent in the better conducting edge, even when the flux-driven supercurrent at the less conducting edge is maximal. This will result in a non-zero net supercurrent flowing across the junction, at all values of magnetic flux. In the interference pattern, it will show up as a lifting of the minimum $\mathbf{I}_c$ [7,8]. Furthermore, in the case of supercurrent flowing only in one of the edges the period of the oscillations will increase significantly, reflecting the confined width of the supercurrent and the small effective area of flux penetration.

The fact that the interference of the BLG junctions drops to zero at half integer values of flux (see Fig. 2c, Supplementary Figure 2c), indicates that the conductance at the two edges is very similar, and that the gate electrode profile does not have a significant effect on the edge modes observed.

To test the electrostatic doping scenario in the Hall bar devices, we compared top gates terminated at the edge of bilayer graphene (see Fig. 4a, main text), or extend far beyond the bilayer (see Supplementary Figure 5a). For both types of devices the sub-gap resistance at high **D** was measured and similar saturation of *R* was observed (Supplementary Figure 5b). It indicates again that the edge profile and the resulting external doping is not significant in these devices.

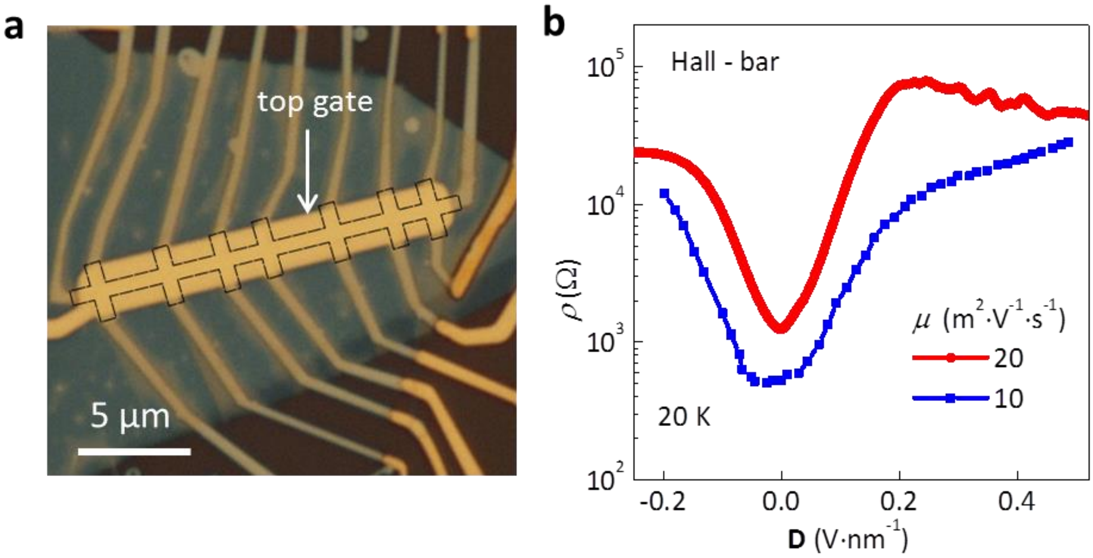

Supplementary Figure 5|Sub-gap resistivity of BLG with the top gate extending above the edges. a, Optical image of the device. Additional hBN cover-layer was placed, enabling the extension of the top gate away from the BLG edges (marked by dashed black line). **b,** Resistivity ρ as a function of the displacement field measured at neutrality point for the device shown in **a** (blue curve). The exponential increase in resistivity is dumped above **D** ≈0.2V·nm^{-1}, where ρ becomes comparable to the quantum of resistance. Devices with gate electrode terminated at the edge (red curve) show a more pronounced saturation, presumably owing to the higher mobility achieved.

To test the effect of electrostatic doping due to uneven distance from the top and bottom gates, we measured a device with the two gates separated by the same distance (Supplementary Figure 6). Again, we find the resistance to saturate at high displacement fields.

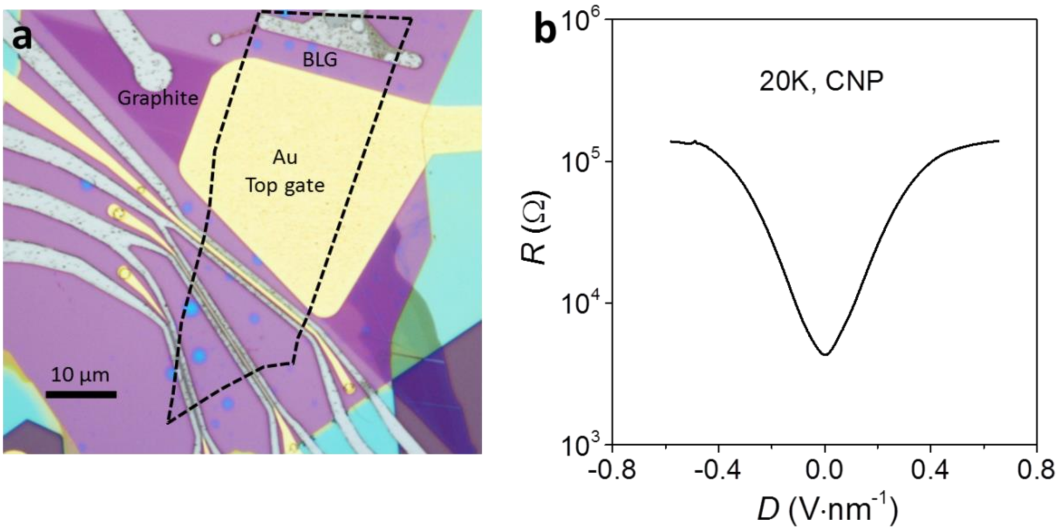

Supplementary Figure 6│Sub-gap resistivity of BLG with even distance from the top and bottom gates. a, Optical image of the device. Graphite flake is used as a bottom gate with equally thick hBN spacers. **b,** Resistance R as a function of the displacement field measured at neutrality point for the device shown in **a**, showing the same saturation at high **D**.

Lastly, we point to the experiments on the gapped monolayer graphene discussed in the main text. There we compare Josephson junctions made using the same fabrication procedures and geometries (including the thickness of the dielectric materials) but for non-aligned (non-gapped) and aligned (gapped) devices. Any inhomogeneity in the external doping should be essentially the same for the two cases. After testing more than 10 non-gapped monolayers[4] and 4 gaped (hBN-aligned) junctions we note that no edge current enhancement was observed in any of the former, while clear edge-dominant currents where observed in all the latter. Here, to avoid the case of the edge-modes being masked by the bulk currents, we examined different aspect-ratios of un-gapped junction with different normal state resistance above the Nb transition temperature or at currents above $\mathbf{I}_c$ (see Fig. 3d,f). We note that no sign of edge currents was found even when the normal state resistance exceeds the resistance where edge-dominant transport was observed in the gapped graphene. It points again to the crucial role of the gap in supporting the enhanced edge conductivity rather than external doping mechanism.

Supplementary Note 5, On-off ratio in gapped bi-layer graphene.

Achieving high on-off ratio in gapped graphene devices is a focus of intense research driven by the practical requirements of electronic applications like field effect transistors (FET)[9]. Owing to the ballistic transport over micron length scales in pristine graphene and BLG at room temperature, the "on" state resistance is mostly determined by the metal-graphene interface resistance, which can be as low as 35 Ohm×µm[4].

The "off" state resistance is usually determined by the size of the gap and the device inhomogeneities. As has been discussed in the main text, for sufficiently clean bilayer graphene devices the edge conductance limits the sub-gap R to the order of the quantum resistance. In the Josephson junction FET geometry for example, the on-off ratio is limited to 10^2 at $\mathbf{D}$=0.2V·nm^{-1} and saturates for higher displacement fields. In contrast, for the edgeless Corbino geometry the highly resistive "off" state is recovered. Here we demonstrate on-off ratio ≈10^4 (at 20K), achieved already

at **D**=0.2V·nm^{-1} owing to the high device homogeneity. Importantly the “off” resistance is limited only by the device quality and the achievable **D**.

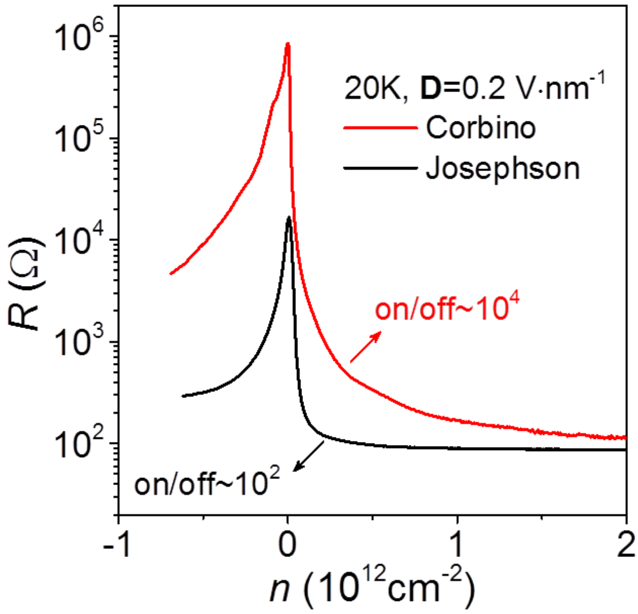

Supplementary Figure 7| On-off ratios in Corbino and in the field effect transistor (FET) geometry. The resistance of the device in the FET geometry (3.5μm wide, 0.4μm long) changes by only 2 orders of magnitude, due to the edge-conductance at the charge neutrality point. In the case of the “edge-less” Corbino geometry, R changes by over 4 orders of magnitude already at **D** =0.2V·nm^{-1}.

Supplementary References

1 Oostinga, J. B., Heersche, H. B., Liu, X. L., Morpurgo, A. F. & Vandersypen, L. M. K. Gate-induced insulating state in bilayer graphene devices. *Nature Materials* **7**, 151-157 (2008).

2 Zhang, Y. *et al.* Direct observation of a widely tunable bandgap in bilayer graphene. *Nature* **459**, 820-823 (2009).

3 McCann, E. Asymmetry gap in the electronic band structure of bilayer graphene. *Phys Rev B* **74**,161403 (2006).

4 Ben Shalom, M. *et al.* Quantum oscillations of the critical current and high-field superconducting proximity in ballistic graphene. *Nat Phys* **12**, 318-U151 (2016).

5 Woessner, A. *et al.* Near-field photocurrent nanoscopy on bare and encapsulated graphene. *Nature communications* **7**, 10783 (2016).

6 Silvestrov, P. G. & Efetov, K. B. Charge accumulation at the boundaries of a graphene strip induced by a gate voltage: Electrostatic approach. *Phys Rev B* **77**, 155436 (2008).

7 Dynes, R. C. & Fulton, T. A. Supercurrent Density Distribution in Josephson Junctions. *Phys Rev B* **3**, 3015 (1971).

8 Hart, S. *et al.* Induced superconductivity in the quantum spin Hall edge. *Nat Phys* **10**, 638-643 (2014).

9 Schwierz, F. Graphene transistors. *Nature Nanotechnology* **5**, 487-496 (2010).
